# Detection of *PIGO*-Deficient Cells Using Proaerolysin: A Valuable Tool to Investigate Mechanisms of Mutagenesis in the DT40 Cell System

**DOI:** 10.1371/journal.pone.0033563

**Published:** 2012-03-12

**Authors:** Jun Nakamura, Husamettin Gul, Xu Tian, Scott J. Bultman, James A. Swenberg

**Affiliations:** 1 Department of Environmental Sciences and Engineering, University of North Carolina, Chapel Hill, North Carolina, United States of America; 2 Department of Pharmacology and Toxicology, Gulhane Military Academy of Medicine, Etlik, Ankara, Turkey; 3 Department of Genetics, University of North Carolina, Chapel Hill, North Carolina, United States of America; 4 Curriculum in Toxicology, University of North Carolina, Chapel Hill, North Carolina, United States of America; Universita' di Milano, Italy

## Abstract

While isogenic DT40 cell lines deficient in DNA repair pathways are a great tool to understand the DNA damage response to genotoxic agents by a comparison of cell toxicity in mutants and parental DT40 cells, no convenient mutation assay for mutagens currently exists for this reverse-genetic system. Here we establish a proaerolysin (PA) selection-based mutation assay in DT40 cells to identify glycosylphosphatidylinositol (GPI)-anchor deficient cells. Using PA, we detected an increase in the number of PA-resistant DT40 cells exposed to MMS for 24 hours followed by a 5-day period of phenotype expression. GPI anchor synthesis is catalyzed by a series of phosphatidylinositol glycan complementation groups (PIGs). The *PIG-O* gene is on the sex chromosome (Chromosome Z) in chicken cells and is critical for GPI anchor synthesis at the intermediate step. Among all the mutations detected in the sequence levels observed in DT40 cells exposed to MMS at 100 µM, we identified that ∼55% of the mutations are located at A:T sites with a high frequency of A to T transversion mutations. In contrast, we observed no transition mutations out of 18 mutations. This novel assay for DT40 cells provides a valuable tool to investigate the mode of action of mutations caused by reactive agents using a series of isogenic mutant DT40 cells.

## Introduction

Research on gene function and regulation is of paramount importance for investigations into the causes of many diseases such as cancer. One very effective approach investigators have been using involves the reverse genetic technique making use of cell lines like the DT40 line which is derived from a chicken B-lymphocyte progenitor. DT40 cells were originated by making use of an avian leucosis virus to induce a bursal lymphoma and the cell line is considered to be isogenic [1]. The DT40 cell line has distinguished itself as being highly valuable as a higher eukaryotic model that exhibits a high ratio of targeted to random integration of transfected DNA [2], [3]. This ease of genetic manipulation, along with the fact that DT40 mutants have been observed to display a strong phenotypic resemblance to murine mutants in DNA recombination and repair [4], has been the foundation that has created a steadily increasing interest in the usage of the DT40 cell line in genetic investigations into immunoglobulin diversification, DNA repair, DNA damage response, chromosome segregation, RNA metabolism and cell signaling [3]–[7].

One downside to the DT40 cell system, however, is that for the last two decades there has been no convenient mutation assay to investigate mechanisms of mutagenicity caused by exogenous reactive agents. One approach to detect mutations in mammalian cells is based on the endogenous gene, hypoxanthine-guanine phosphoribosyltransferase (*HPRT*), located on the X chromosome [8]. This method has been extensively utilized in toxicology [9], [10]. The *HPRT* mutants from cultured cells and tissues of animals exposed to mutagens are resistant to the toxic nucleoside 6-thioguanine, which allows the selection of mutant cells. Thus, mutation rates and mutational sequences in the *HPRT* locus can be analyzed in 6-thioguanine-resistant cells. While the *HPRT* mutation assay has gained popularity in mutational analysis in mammalian cells, this assay is difficult to perform in DT40 cells due to the presence of the *HPRT* gene on the autosomal chromosome (chromosome 4) in chicken cells instead of the sex chromosome.

**Figure 1:**
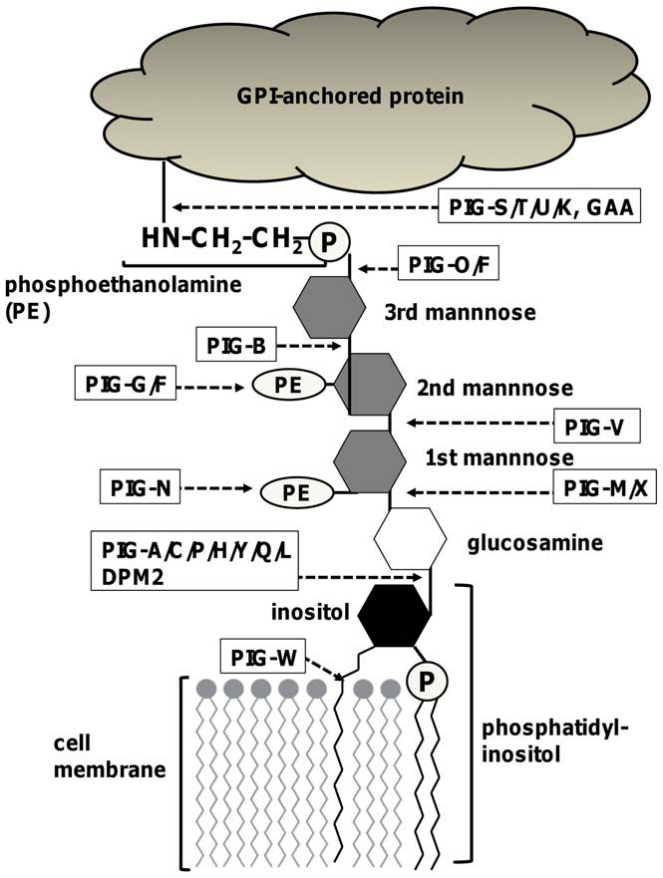
Biosynthesis of the glycosylphosphatidyl inositol (GPI)-anchored protein. Synthesis of GPI-anchored proteins involves multiple reaction steps. Briefly, the first step of GPI anchor biosynthesis is catalyzed by a multi-subunit GPI-*N*-acetylglucosaminyltransferase comprised of at least 6 different proteins (PIG-A, PIG-C, PIG-H, PIG-P, PIG-Q, PIG-Y). In addition, DPM2 appears to regulate this first step, followed by de-*N*-acetylation by the PIG-L. PIG-W then attaches an acyl chain to form glucosamine-(acyl)PI. In the next step, three mannose (Man) residues are added sequentially to glucosamine-(acyl)PI, generating Man-Man-Man-glucosamine-(acyl)PI by PIG-M/PIG-X complex, PIG-V, and PIG-B. After the Man-1 and Man-2 conjugation, PIG-N adds ethanolamine phosphates (EtNP) to the Man-1. In the final step of GPI anchor synthesis, PIG-O/PIG-F and PIG-G/PIG-F complexes attach EtNP to the Man-3 and Man-2, respectively, to generate the mature GPI anchor protein.

Recently, a novel mutation assay was developed in rats, making use of the endogenous phosphatidylinositol glycan complementation group A (*Pig-a*) gene for red blood cells and T-lymphocytes [11], [12]. The *PIG-A* gene has several characteristics that make it well suited for the detection of somatic cell mutations. Although over 20 *PIG* genes, including *PIG-A*, were identified for GPI anchor synthesis (Figure 1), only the *PIG-A* gene is located on the X chromosome in mammalian cells (Table 1) [13]. As with the *HPRT* mutation, a single mutation on the *PIG-A* gene results in an altered cell phenotype in mammalian cells. *PIG-A* codes for an enzyme that, along with five other cores and one extra subunit (PIG-C/-H/-P/-Q/-Y and DPM2), is involved in the first step of GPI anchor synthesis (Figure 1) [13]–[17]. The functions of all six core subunits are essential or nearly essential for GPI anchor synthesis [17]. GPI anchors tether specific protein markers to the surface of various types of cells (e.g., hematopoietic cells) [18], [19]. Thus, mutations in the *PIG-A* gene could disrupt GPI anchor synthesis which, in turn, would cause a deficiency in GPI-anchored proteins. Lack of the anchor or GPI-anchored proteins can be detected and quantified using a flow cytometer. Furthermore, proaerolysin (PA) has been successfully utilized in spleen T-cells to select for GPI anchor-deficient cells in the *Pig-a* mutation clonal assay [20]. PA is a protoxin form of aerolysin, which is a channel-forming microbial toxin [21]–[23]. After binding to GPI at the cell surface, PA is proteolytically cleaved into aerolysin, which ultimately forms transmembrane pores [24] and causes cell death. Mutations in the *PIG-A* gene abrogate GPI anchor synthesis and deplete GPI anchors at the surface of cells. Therefore, the GPI anchor-deficient mutant cells become resistant to aerolysin-induced cytotoxicity. In addition to determining the frequency of *PIG-A* gene mutations, it is possible to determine the *PIG-A* mutation spectrum through sequencing following RT-PCR of mRNA extracted from cells resistant to PA [20]. However, the *PIG-A* gene is also located on an autosome in DT40 cells (Table 1). DT40 cells were established from B lymphocytes of female chickens having female heterogamety with Z and W sex chromosomes. Both *PIG-G* and *PIG-O* genes are involved in GPI anchor synthesis; however, in contrast to the *PIG-A* gene, both are located on chromosome Z. Modification of the GPI anchor by PIG-G is not essential for the function of the GPI anchor at the cellular level [25]. However, a complex of PIG-O and PIG-F catalyzes an attachment of phosphoethanolamine to the third mannose of the GPI anchor in the final step (Figure 1) immediately before attachment of the GPI anchor and cell surface protein. Since the phosphoethanolamine attached to the third mannose is the location where the surface protein binds, the *PIG-O* gene product is nearly essential for GPI anchor synthesis [26]. In the present study, we established a new *PIG-O* gene mutation assay to analyze the mutagenicity of reactive chemicals in DT40 cells through PA selection.

**Table 1:** Chromosome location of GPI anchor synthesis genes.

	chicken	human	rat
**PIG-A**	Chr 1	Chr X	Chr X
**PIG-C**	Chr 8	Chr 1	Chr 13
**PIG-P**	Chr 1	Chr 21	Chr 11
**PIG-H**	Chr 5	Chr 14	Chr 6
**DPM2**	Chr 17	Chr 9	Chr 3
**PIG-Y**	N.I.	Chr 4	Chr 4
**PIG-Q**	Chr 14	Chr 16	Chr 10
**PIG-L**	Chr 19	Chr 17	Chr 10
**PIG-W**	Chr 19	Chr 17	Chr 10
**PIG-M**	Chr 3	Chr 1	Chr 13
**PIG-X**	Chr 9	Chr 3	Chr 11
**PIG-V**	N.I.	Chr 1	Chr 5
**PIG-N**	Chr 2	Chr 18	Chr 13
**PIG-B**	N.I.	Chr 15	Chr 18
**PIG-F**	N.I.	Chr 2	Chr 6
**PIG-O**	Chr Z	Chr 9	Chr 5
**PIG-G**	Chr Z	Chr 4	Chr 14
**PIG-S**	Chr 19	Chr 17	Chr 10
**PIG-T**	Chr 20	Chr 20	Chr 3
**PIG-U**	Chr 20	Chr 20	Chr 3
**PIG-K**	Chr 8	Chr 1	Chr 2
**GAA1**	N.I.	Chr 8	Chr 7

Chr: Chromosome; N.I.: not identified.

## Results and Discussion

### Cytotoxicty of PA in DT40 cells

**Figure 2:**
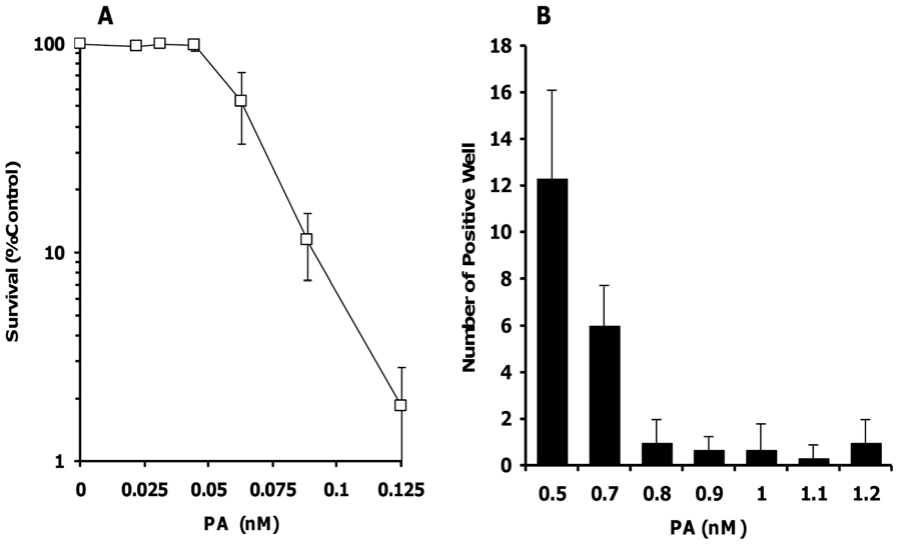
Cell survival after PA exposure. (**A**) In a low cell density experiment using a 24-well plate (2.5×10^3^ cells/250 µL/well), DT40 cells were exposed to PA (0.0221–0.125 nM). After a three-day cultivation, cell viability was determined by XTT. Each point represents the mean and S.D. (bars) from three independent experiments. (**B**) In a high cell density experiment using a 96-well plate (4×10^4^ cells/50 µl/well), DT40 cells were exposed to PA (0.5–1.2 nM). After a seven-day incubation, colony formation was scored visually using an inverted microscope. Each point represents the mean and S.D. (bars) from three independent experiments.

A concentration of PA suitable for the selection of GPI anchor-deficient DT40 cells was established using intact DT40 cells. In a low cell density experiment using a 24-well plate (2.5×10^3^ cells/250 µL/well), the DT40 cells were exposed to PA at 0.125 nM or lower for three days. PA caused marked cell death in DT40 cells at 0.125 nM (Figure 2A). Furthermore, in a high cell density experiment using a 96-well plate (4×10^4^ cells/50 µl/well), nearly all cells were killed by PA treatment at 0.8 nM or higher during a 7-day cultivation period (Figure 2B). Based on these results, we chose 1.2 nM PA for the selection of GPI anchor-deficient cells for the rest of the study.

### Characterization of PA^r^ DT40 cells

**Figure 3:**
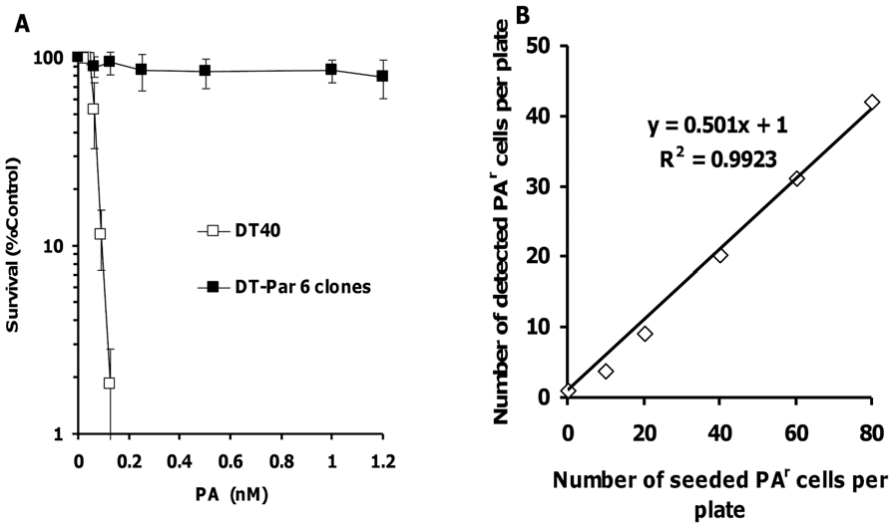
Characterization of PA-resistant (PA^r^) DT40 cells and validation of PA selection-based GPI anchor-deficient cell detection assay. (**A**) In a low cell density experiment using a 24-well plate (2.5×10^3^ cells/250 µL/well), the intact DT40 cells and six different clones of DT40 cells that survived from the first PA treatment at 1.2 nM were exposed to PA (0.0221–1.2 nM). After a three-day cultivation, cell viability was determined by XTT. Each point represents the mean and S.D. (bars) from three independent experiments for DT40 cells and single experiment for six different clones of DT40 cells resistant to PA. (**B**) Using one of the PA^r^ clones used for Figure 3A, different numbers of PA^r^ cells (0 to 80 cells/plate) were seeded onto 96-well plates containing intact DT40 cells (40×10^3^ cells/well) to validate the accuracy of the PA selection step of the assay. The cells were exposed to PA at 1.2 nM. After a seven-day incubation, colony formation was scored visually using an inverted microscope. Plating efficiency was also determined using PA^r^ cells.

In order to confirm whether the DT40 cells that survived PA exposure were indeed PA^r^ cells, we re-challenged the cells to different concentrations of PA. Compared to intact DT40 cells, six different clones of DT40 cells that survived the first PA treatment at 1.2 nM were resistant to cell death induced by PA (Figure 3A), indicating that accurate selection of PA^r^ DT40 cells is feasible using 96-well plates. Using one of the PA^r^ clones, we seeded different numbers of PA^r^ cells (0 to 80 cells/plate) onto 96-well plates containing intact DT40 cells (40×10^3^ cells/well) to validate the accuracy of the PA selection step of the assay. We detected a linear relationship between expected and observed frequencies of PA^r^ cells (R^2^ = 0.99, y = 0.501x+1) (Figure 3B).

### Optimization of detection of GPI anchor-deficient DT40 cells exposed to MMS

**Figure 4:**
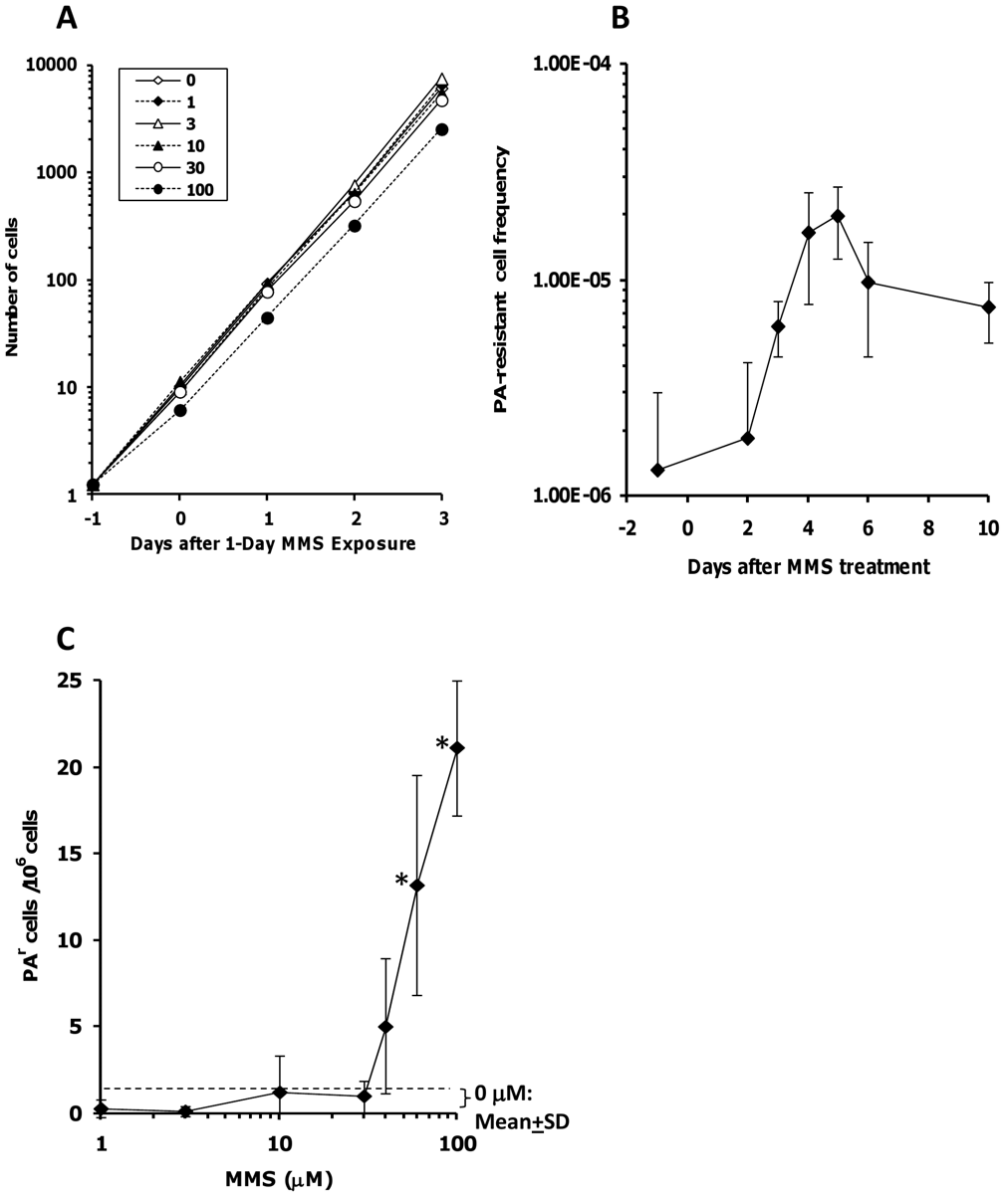
Frequency of GPI anchor-deficient DT40 cells exposed to MMS. (A) The effect of 24-hour MMS exposure at 0, 1, 3, 10, 30, or 100 µM on cell growth of DT40 cells was evaluated over three days after MMS exposure. The cell growth assay was performed during/after MMS treatment. Each point represents the mean and S.D. (bars) from three independent experiments. (B) The length of the phenotype expression period in the PA^r^ DT40 cells was optimized after 100 µM MMS treatment for 24 hours. The frequency of PA^r^ DT40 cells was determined before and after MMS treatment. Each point represents the mean and S.D. (bars) from at least three independent experiments. (C) Frequency of PA^r^ DT40 cells was determined after exposure to MMS at different concentrations. DT40 cells were exposed to MMS at 0, 1, 3, 10, 30, 40, 60, and 100 µM for 24 hours. The cells were further cultured for five days in fresh medium without MMS. The frequency of PA^r^ DT40 cells was determined for each group. Each point represents the mean and S.D. (bars) from at least three independent experiments. P<0.05. Discontinued line shows the mean and S.D. of mutational frequency in the control samples (0.5±0.8 mutants/10^6^ cells).

MMS is a well-studied mutagen; therefore, we utilized this reactive agent to optimize the PA-selection-based GPI anchor-deficient cell detection assay. The effect of 24-hour MMS exposure at 100 µM on cell growth was first evaluated over three days after MMS exposure. While the first 24-hour incubation with 100 µM MMS decreased the growth rate to ∼80% compared to the control, the cell growth rate recovered to control level after replenishing the medium without MMS (Figure 4A). It has been well established that, following a mutation at the *HPRT* locus, time is required before the new thioguanine-resistant phenotype is completely expressed [27]. We next optimized the length of the phenotype expression period in the PA^r^ DT40 cells after MMS treatment. The length of expression time showing a maximum mutant frequency depends on segregation of the mutant allele from the normal allele by cell proliferation; loss of the original, normal gene activity due to dilution or degradation of wild-type protein; and loss of original, wild-type mRNA due to its dilution or degradation [27]. An increase in PA^r^ cells was detected 3 days after MMS treatment, and the number of PA^r^ cells reached nearly maximum at 5 days after MMS treatment (Figure 4B). This indicates that a 4- to 5-day cultivation is required for full phenotypic expression (GPI anchor deficiency) following MMS treatment. We also detected a decrease in the frequency of PA^r^ DT40 cells after a 6-day expression period. This reduction appears to be due to slower proliferation rates of mutant than non-mutant cells; therefore, propagation longer than five days may lead to further decreases in the PA^r^ cell frequencies. Based on this time-course experiment, we chose a 24-hour MMS exposure plus a 5-day phenotype expression period for most of the experiments in this study. While the PA^r^ cells were allowed to grow for 5–7 days in the presence of PA before identifying resistant cells, small colonies of PA^r^ cells were usually detectable as early as 3 days after the PA treatment.

### Frequency of GPI anchor-deficient DT40 cells exposed to MMS at different concentrations

MMS has recently been evaluated for its low dose effects in the *HPRT* and *TK* genes in the AHH-1 human lymphoblastoid cell line [28]. MMS exposure for 24 hours caused a nonlinear (hockey-stick-shaped dose-response) curve containing a range of non-mutagenic low doses in both mutation assays. We next addressed whether the appearance rate of PA^r^ cells after MMS treatment showed a non-linear dose response curve in DT40 cells. DT40 cells were exposed to different concentrations of MMS to determine an increase in the rate of PA^r^ cell formation. Cell growth inhibition was observed at 80 or 100 µM after 24-hour MMS exposure, but not at 60 µM or lower (Figure 4A and data not shown). We detected the hockey stick-like dose-response curve in the incidence of PA^r^ cells (Figure 4C). These results indicate that a PA-based selection is a useful tool to detect GPI anchor synthesis gene mutations in DT40 cells. When we compared different mutation assays in different cell lines, we recognized that the PIG-O mutation assay in DT40 cells required slightly higher concentrations of MMS compared to the HPRT mutation assay in AHH-1 cells [28]. This difference could be due to the variation of the functionality of various DNA repair pathways against DNA lesions caused by MMS.

### PIG-O gene is mutated in PA^r^ DT40 cells

Paroxysmal nocturnal hemoglobinuria (PNH) is an acquired hematopoietic stem-cell disorder resulting from the clonal expansion of hematopoietic cells with somatic mutations in the X-linked *PIG-A* gene in humans. The affected cells with *PIG-A* gene mutations are deficient in GPI-anchored proteins. PNH is frequently associated with hemolytic anemia, a hypercoagulable state, and bone marrow failure [29]. Many proteins on the eukaryotic cell surface are anchored by GPI [30]. While the *PIG-A* gene is located in the autosome in DT40 cells, there is a single copy of the *PIG-O* gene located on the Z chromosome in the cells derived from the female chicken. The chicken *PIG-O* gene codes for a protein that contains 1145 amino acids (XP_001232870). The percent amino acid sequence identity of the chicken PIG-O with the human PIG-O (1089 amino acids, NP_116023) is 94%. To address whether PA^r^ DT40 cells possess a *PIG-O* gene mutation, we next sequenced *PIG-O* cDNA in both normal DT40 cells and DT40 cells resistant to PA. Figure S1 shows the comparison between the *PIG-O* cDNA sequences found in DT40 cells and those found in chicken (XM_001232869). The cDNA sequence in parental DT40 cells was used as a reference for determining DNA alterations in PA^r^ DT40 cells. Among all the mutations detected in the sequence levels observed in DT40 cells exposed to MMS, we identified that ∼55% of the mutations are located at A:T sites, including a high frequency of A to T trans-version mutations and three frameshift mutations (Table 2). In contrast, there were no transition mutations out of the 18 mutations. We also observed three clones with deletions ranging from 20 to 204 bases and three clones with insertions ranging from 2 to 6 bases. When we compared our results in the mutational spectrum of the PIG-O gene in DT40 cells and the previously published spectrum of the *hprt* gene in CHO cells, there was a drastic difference in the mutational spectrum caused by MMS exposure. We detected no G/C→A/T transition mutations in our study whereas predominant G/C→A/T transition mutations at the *hprt* gene have been frequently reported in Chinese hamster fibroblast/ovary cells [31], [32]. This can be explained by a O^6^-methylguanine-DNA-methyltransferase deficiency in these Chinese hamster fibroblast/ovary cells, which leads to the accumulation of mutagenic O^6^-methylguanine. In contrast, the high frequency of A/T→T/A trans-version mutations in the PIG-O gene in DT40 cells is in agreement with the mutational spectrum of target genes obtained from in vivo exposure to MMS. For example, mutations with a high frequency of A/T→T/A trans-versions have been demonstrated at the *hprt* gene in splenic T-lymphocytes from N-methylpurine-*DNA* glycosylase knockout mice exposed to MMS and the *vermilion* locus of *Drosophila melanogaster* exposed to MMS [33]–[35]. The A/T→T/A trans-version mutations found in MMS exposure-derived PA^r^ DT40 cells appear to be due to N3-methyladenine or base excision repair intermediates, such as abasic sites and single strand breaks originated from N3-methyladenine [36], [37]. Although more work needs to be done to characterize the spectrum of spontaneous mutations (Table 3), most of the *PIG-O* gene mutations in DT40 cells exposed to MMS represent mutations derived from DNA damage caused by MMS due to the increase of mutation frequency by more than 45 times over the spontaneous mutation rate (Figure 4C). Furthermore, it is worthwhile to mention that our preliminary study showed a high background of PIG-O mutations in some of DNA repair gene-deficient DT40 cells (e.g., XPA-deficient cells), confirming the important value of this new mutation assay in DT40 cell system. It has been previously reported that PIG-O and PIG-F act together in transferring phosphoethanolamine to the third mannose during GPI anchor synthesis in CHO cells [26]. In the same study, the *Pig-o* gene mutant cells showed a pronounced decrease, but not a complete loss, in the expression of Thy-1, a GPI-anchored protein, in CHO cells. The *Pig-o* gene mutant CHO cells also expressed no detectable CD59 and only 10% of normal levels of decay-accelerating factor (both CD59 and decay-accelerating factor are GPI-anchored proteins) [38], indicating that *Pig-o* is involved in and nearly essential for GPI anchor biosynthesis [26]. We attempted to detect *PIG-O* mutants using fluorescent-tagged proaerolysin (FLAER)-coupled FACS analysis; however, the separation between FLAER-positive and negative DT40 cells was not accomplished in our study. This could be due to partial expression of GPI-anchored proteins on the membrane in *PIG-O* mutants as described above. Therefore, we chose the PA selection-based method instead of the FACS-based analysis to analyze the frequency of GPI-anchor deficient DT40 cells with high specificity. As described above, PA selection-based GPI-deficiency cell analysis demonstrated a reasonable dose-response relationship between MMS concentrations and the frequency of GPI-deficient cells, indicating that this assay is a useful tool for characterizing mutation events in the DT40 cell system.

**Table 2:** Mutational spectrum of *PIG-O* gene in DT40 cells exposed to MMS at 100 µM.

Mutation (cDNA position)	Target sequence (5′-3′)	Amino acid change (codon)
Transversion		
AT to TA (840)	CCA(**T**)GGT	His to Gln (280)
AT to TA (2009)	GCC(**T**)GAG	Leu to Gln (670)
AT to TA (2011)	CTG(**A**)GAA	Arg to stop (671)
AT to TA (2332)	GCA(**A**)AGG	Lys to stop (778)
AT to TA (2941)	TCC(**A**)AGA	Lys to stop (981)
AT to CG (689)	ACC(**A**)CTG	His to Pro (230)
AT to CG (950)	ACC(**T**)GGT	Leu to Arg (317)
GC to CG (2771)	ACT(**G**)GAA	Trp to Ser (924)
GC to TA (2911)	TGT(**G**)AGA	Glu to stop (971)
Frameshift		
+A (1915)	CTT(*)CTG	
−T (241)	GAA(**T**)TTG	
−A (941)	CCC(**A**)GGT	
Insertion/Deletion		
+6 bases (1092)	CAA(**GCAGTT**)GCA	
+2 bases (2786)	TTG(**CG**)TGG	
+4 bases (2873)	CAG(**GTAT**)TTG	
−145 bases (497–641)	GAA(**GAA—GAA**)CAG	
−20 bases (1746–1765)	AGC(**TGA—CAT**)TCC	
−204 bases (2669–2872)	CTG(**AGC—CAG**)TTG	

**Table 3:** Spontaneous mutational spectrum of *PIG-O* gene in DT40 cells.

Mutation (cDNA position)	Target sequence (5′-3′)
Deletion	
−171 bases (923–1093)	AGG(**AGC—AGC**)AGT
−4 bases (1048–1051)	GTG(**TCTG**)AAG
−4 bases (1093–1096)	AAG(**CAGG**)TGG
−4 bases (1092–1095)	CAA(**GCAG**)GTG

## Materials and Methods

### Materials

Penicillin/streptomycin and methyl methanesulfonate (MMS) were obtained from Sigma (St. Louis, MO). Trizol, *Taq* DNA polymerase, RPMI 1640 culture medium and chicken serum were acquired from Invitrogen (Carlsbad, CA). Fetal bovine serum (FBS) was obtained from PAA laboratories Inc (Etobicoke, Canada). iScript cDNA synthesis kit and PCR primers were acquired from Bio-Rad Laboratories (Hercules, CA) and Thermo Fisher Scientific (Waltham, MA), respectively. Wizard SV Gel/PCR Clean-Up System and PA were purchased from Promega (Madison, WI) and the University of Saskatchewan (Saskatoon, Canada), respectively.

### Cell growth assay after MMS exposure

DT40 cells [6] were cultured in a manner previously reported [6], [7]. We performed two different types of cell survival assays:

#### Low cell density experiment

One of the cell survival assays was conducted as described previously for the DNA damage response analysis [6], [7]. Briefly, suspended DT40 cells (∼2.5×10^3^ cells per 250 µL per well) were seeded into 24-well plates, exposed to either PA or MMS, and allowed to divide for 7 to 8 cycles (approximately 3 days). After cultivation, cell viability was determined by the XTT assay [6], [7].

#### High cell density experiment

We performed a cell growth assay during/after MMS treatment. The assay was performed under the exposure conditions used for the PA-selection-based GPI anchor-deficient cell detection assay described below. Briefly, suspended DT40 cells (1. 25×10^6^ cells/10 ml) were seeded into 10-cm Petri dishes, exposed to MMS, and allowed to cultivate for 24 hours. The number of viable cells was quantitated by the trypan blue exclusion assay. The culture medium was replenished and the cells (1. 25×10^6^ cells/10 ml) were further incubated for 24 hours. This cycle was repeated for 5 days.

### PA-selection-based GPI anchor-deficient cell detection assay

The overall scheme of this assay is shown in Table 4. The cells were maintained at a concentration of 0.125 to 1.5×10^6^/mL. Since the background PA-resistant (PA^r^) cell population was low, we directly used DT40 cells for the assay without re-populating with PA-sensitive DT40 cells in this study. Cell suspension at 1.25×10^6^ cells/10 mL was exposed to MMS at different concentrations for 24 hours at 39.5°C, 5% CO_2_. The cells were then washed to remove the MMS and were resuspended in 10-mL fresh medium (1.25×10^6^ cells/10 mL). The culture medium was replenished daily to maintain appropriate cell concentrations as described above. The 96-well plates were then loaded with 4×10^4^ cells/50 µL/well containing 1.2 nM PA (2 plates per sample). Nonselection plates were prepared by inoculating 2 target cells and 4×10^4^ lethally lactic acid-pre-treated (50 mM lactic acid for 30 min at 39°C) DT40 cells as feeder cells in 50 µl medium in each well to determine the plating efficiency in the absence of PA treatment (2 plates per sample). All plates were incubated for approximately 7 days at 37°C, in 5% CO_2_, and in a humidified atmosphere. Subsequent colony formation was scored visually using an inverted microscope to determine the mutation frequency of each dose, calculated as described by Furth et al. [39].

**Table 4:** Scheme of *PIG-O* mutation assay in DT40 cells.

1. Seed cells into a 10-cm dish at 1.25 million/10 ml.
2. Expose cells to MMS for 24 hours.
3. Replenish medium with cells at 1.25 million/10 ml everyday after MMS exposure.
4. Seed approximately 40,000 cells/well in 96-well plate at five days after MMS treatment.
5. Treat cells with proaerolysin at 1.2 nM.
6. Incubate cells for seven days.
7. Count colonies under the microscope.

### Reverse transcription-polymerase chain reaction

RNA was prepared using Trizol reagent and was reverse-transcribed using a mixture of random hexamers and Oligo d(T) according to standard procedures. Primers for *PIG*-O PCR were designed using Primer-BLAST (National Center for Biotechnology Information) based on a predicted chicken *PIG-O* cDNA sequence (XM_001232869.1). cDNA (150 ng/25 µL) was PCR-amplified in 20 mM Tris (pH 8.4), 50 mM KCl, 1 mM MgCl_2_, 0.1 mM dNTPs, 0.5 µM primers, and 2.5 U *Taq* polymerase. The cycling parameters were 94°C for 30 sec, 60°C for 30 sec, and 72°C for 1 min for 35 cycles. The sequences of primers 1–6 are shown in Table 5. The PCR products were purified on Wizard SV Gel and PCR Clean-Up System, and the *PIG-O* coding sequence was determined in the Genome Analysis Facility (University of North Carolina at Chapel Hill). Mutations were identified by a comparison between the *PIG-O* cDNA sequence of each mutant clone and the wild-type sequence obtained from intact parental DT40 cells (Figure S1).

**Table 5:** Primers for the RT-PCR and Sequencing of Chicken *PIG-O* cDNA.

Primers	Position of primers^a^	Nucleotide sequence (5′→3′)
1F	-48 to -26	GCTGAGAT AGAGCCTTGG GGGCT
1R	450 to 472	GCAGGTTGTCCTCCTGGATCGCA
2F	369 to 388	TGCCACCATGCAGCGCATCA
2R	1287 to 1306	CCCGTGCCTGCCGCAGATAG
3F	1127 to 1146	TGGCTCAGGACCTGCCAGCA
3R	1666 to 1685	AAGGCCAGCCCAAGCCACAC
4F	1593 to 1612	GCATCCCAAGCGAGCCCGTT
4R	2075 to 2094	CAGCCAGCTCCGCACTGCAT
5F	1968 to 1988	CAGTGCCGGCCCTCCGTTTT
5R	2505 to 2524	GGGCTGCCGAGTACACGCTG
6F	2470 to 2489	AGCTGCAGGGCCACAGTTGC
6R	2980 to 2999	GGGGACTCCCGCAGCCTCAT

**^a^**Primer annealing sites relative to the A of the ATG initiation codon (XM_001232869).

### Statistical analysis

A one-way ANOVA, followed by a Dunnett's posthoc test, was used to determine if any of the treatment doses were significantly different from the zero dose in the 24-hour MMS exposure experiment.

## Supporting Information

Figure S1 **Comparison between the** ***PIG-O*** **cDNA sequences found in DT40 cells and those found in chicken (XM_001232869).**
(DOCX)
