# Supplementary material for: Detection of PIGO-Deficient Cells Using Proaerolysin: A Valuable Tool to Investigate Mechanisms of Mutagenesis in the DT40 Cell System
Source: PLoS One. 2012 Mar 12;7(3):e33563. doi: 10.1371/journal.pone.0033563 (PMC3299801; doi:10.1371/journal.pone.0033563)
Supplement: Figure S1 — Comparison between the PIG-O cDNA sequences found in DT40 cells and those found in chicken (XM_001232869). (DOCX) [file pone.0033563.s001.docx]

**Figure S1.**

Top of Form

Bottom of Form

Top of Form

**Reference (Refe):** |XM_001232869.1| PREDICTED: Gallus gallus similar to MGC80777 protein (LOC769584),

mRNA Length=3438

GENE ID: 769584 PIGO | phosphatidylinositol glycan anchor biosynthesis, class O

[Gallus gallus]

Score = 5404 bits (2926), Expect = 0.0

Identities = 2985/3012 (99%), Gaps = 12/3012 (0%)

Strand=Plus/Plus

DT40 1 ATGCAGCGGTGGCCGGTGCTGCTTTTCCTGGCCTGGGTCTGCTTTCTCTTCTTCTCTGGT 60

||||||||||||||||||||||||||||||||||||||||||||||||||||||||||||

Refe 1 ATGCAGCGGTGGCCGGTGCTGCTTTTCCTGGCCTGGGTCTGCTTTCTCTTCTTCTCTGGT 60

DT40 61 ATTGGGCTCTTCATGAGTGGCTTCCTGCTCACCCGTATTGAGCTTGCCAACAGCAGTTCC 120

||||||||||||||||||||||||||||||||||||||||||||||||||||||||||||

Refe 61 ATTGGGCTCTTCATGAGTGGCTTCCTGCTCACCCGTATTGAGCTTGCCAACAGCAGTTCC 120

DT40 121 TGCTCAGACCCACTCGCGCCACCACCGTGGGACAAGCAGAGCCTCCCACCAGGCTCTTGT 180

||||||||||||||||||||||||||||||||||||||||||||||||||||||||||||

Refe 121 TGCTCAGACCCACTCGCGCCACCACCGTGGGACAAGCAGAGCCTCCCACCAGGCTCTTGT 180

DT40 181 TGGGTCCCCCAGCGCTTTCCCAAGGTCGTGCTTGTTATCATCGATGCCCTCCGTTTTGAA 240

||||||||||||||||||||||||||||||||||||||||||||||||||||||||||||

Refe 181 TGGGTCCCCCAGCGCTTTCCCAAGGTCGTGCTTGTTATCATCGATGCCCTCCGTTTTGAA 240

DT40 241 TTTGCCTTGTTTAACCCAGCCAAGGTCAACCCGCTGCCCTATGAGAATAAGCTGAGTTTT 300

||||||||||||||||||||||||||||||||||||||||||||||||||||||||||||

Refe 241 TTTGCCTTGTTTAACCCAGCCAAGGTCAACCCGCTGCCCTATGAGAATAAGCTGAGTTTT 300

DT40 301 CTGCACCACCTGGCAACCTCTCAGCCCCACCATGCCCGCCTCTACCGCTTCCTAGCTGAT 360

|||||||||||||||||||||||||||||||| |||||||||||||||||||||||||||

Refe 301 CTGCACCACCTGGCAACCTCTCAGCCCCACCACGCCCGCCTCTACCGCTTCCTAGCTGAT 360

DT40 361 CCCCCCACTGCCACCATGCAGCGCATCAAGGGCCTCACCACCGGGTCACTGCCTACTTTC 420

||||||||||||||||||||||||||||||||||||||||||||||||||||||||||||

Refe 361 CCCCCCACTGCCACCATGCAGCGCATCAAGGGCCTCACCACCGGGTCACTGCCTACTTTC 420

DT40 421 ATTGATGTGGGCAGTAACTTTGCTACCTATGCGATCCAGGAGGACAACCTGCTGGCACAG 480

||||||||||||||||||||||||||||||||||||||||||||||||||||||||||||

Refe 421 ATTGATGTGGGCAGTAACTTTGCTACCTATGCGATCCAGGAGGACAACCTGCTGGCACAG 480

DT40 481 CTGGTGCAGAATGGAAGAAGAGTGGTCTTCATGGGTGATGATACCTGGGAAGGACTCTTC 540

||||||||||||||||||||||||||||||||||||||||||||||||||||||||||||

Refe 481 CTGGTGCAGAATGGAAGAAGAGTGGTCTTCATGGGTGATGATACCTGGGAAGGACTCTTC 540

DT40 541 CCAAAGAAGTTTTTCCGGTCTTATTTTTTCCCTTCTTTCAATGTGAAGGATCTTCACACT 600

||||||||||||||||||||||||||||||||||||||||||||||||||||||||||||

Refe 541 CCAAAGAAGTTTTTCCGGTCTTATTTTTTCCCTTCTTTCAATGTGAAGGATCTTCACACT 600

DT40 601 GTGGACAATGGGATCTTGCAGTACCTTTATCCAACTGTGAACAGTGGTGAGTGGGACTTG 660

||||||||||| ||||||||||||||||||||||||||||||||||||||||||||||||

Refe 601 GTGGACAATGGTATCTTGCAGTACCTTTATCCAACTGTGAACAGTGGTGAGTGGGACTTG 660

DT40 661 CTGATTGCTCACTTCCTCGGTGTGGACCACTGTGGGCACAAACATGGACCTGACCATCCT 720

||||||||||||||||||||||||||||||||||||||||||||||||||||||||||||

Refe 661 CTGATTGCTCACTTCCTCGGTGTGGACCACTGTGGGCACAAACATGGACCTGACCATCCT 720

DT40 721 GAAATGGCCAAGAAGCTCACTCAGATGAATGAGATGCTCAGGTCCTTGGTGGATCACCTG 780

|||||||||||||||||||||||||||||||||||||||||||||||||||||||||||

Refe 721 GAAATGGCCAAGAAGCTCACTCAGATGAATGAGATGCTCAGGTCCTTGGTGGATCACCTA 780

DT40 781 GGGAATGACACTCTCCTTCTGGTGGCTGGAGACCATGGCATGACAGAGACTGGAGACCAT 840

||||||||||| ||||||||||||||||||||||||||||||||||||||||||||||||

Refe 781 GGGAATGACACGCTCCTTCTGGTGGCTGGAGACCATGGCATGACAGAGACTGGAGACCAT 840

DT40 841 GGTGGTGACAGCCAGAGGGAAGTGAATGCAGCACTGTTCGTGTACAGTAAAACACCTCTG 900

|||||||||||||||||||||||||||||||||||||||||||||||||||||||| ||

Refe 841 GGTGGTGACAGCCAGAGGGAAGTGAATGCAGCACTGTTCGTGTACAGTAAAACACCCCTA 900

DT40 901 TTTGGCACTGATCCTCCTGAGGAGCCTGAGGCCATTCCCCAGGTGAACCTGGTGCCCACT 960

||||||||||||||||||||||||||||||||||||||||||||||||||||||||||||

Refe 901 TTTGGCACTGATCCTCCTGAGGAGCCTGAGGCCATTCCCCAGGTGAACCTGGTGCCCACT 960

DT40 961 GTGGCCCTGCTGCTGGGTGTGCCCATTCCCTACAGTAACATCGGGGAAGTGATGGCTGAG 1020

||||||||||||||||||||||||||||||||||||||||||||||||||||||||||||

Refe 961 GTGGCCCTGCTGCTGGGTGTGCCCATTCCCTACAGTAACATCGGGGAAGTGATGGCTGAG 1020

DT40 1021 CTGTTCTCCGGGGATGGTGACACTGTGTCTGAAGCCTTGCAGCAGCTCTTGGTTTATCAC 1080

||||||||||||||||||||||||||||||||||||||||||||||||||||||||||||

Refe 1021 CTGTTCTCCGGGGATGGTGACACTGTGTCTGAAGCCTTGCAGCAGCTCTTGGTTTATCAC 1080

DT40 1081 ATCAATGCCAAGCAGGTGGACCGCTTCCTGCATTCGTACTCACTGGTGGCTCAGGACCTG 1140

||||||||||||||||||||||||||||||||||||||||||||||||||||||||||||

Refe 1081 ATCAATGCCAAGCAGGTGGACCGCTTCCTGCATTCGTACTCACTGGTGGCTCAGGACCTG 1140

DT40 1141 CCAGCAGAGCAGCTCCAGCACCTGCAGGAGCTCTTCTCCAGTGCTGTAGAGGAGCACGTT 1200

||||||||||||||||||||||||||||||||||||||||||||||||||||||||||||

Refe 1141 CCAGCAGAGCAGCTCCAGCACCTGCAGGAGCTCTTCTCCAGTGCTGTAGAGGAGCACGTT 1200

DT40 1201 CAGCTCTTGGCCCAGGTGCAGAGGGCAATGCTGGTGTCTCCGGAATTGGAGTCCAAGCTC 1260

||||||||||||||||||||||||||||||||||||||||||||||||||||||||||||

Refe 1201 CAGCTCTTGGCCCAGGTGCAGAGGGCAATGCTGGTGTCTCCGGAATTGGAGTCCAAGCTC 1260

DT40 1261 AGAAGCCTCATCAGCCGCTTCCAGCTCTATCTGCGGCAGGCACGGGCTGTGTGCACCCAG 1320

||||||||||||||||||||||||||||||||||||||||||||||||||||||||||||

Refe 1261 AGAAGCCTCATCAGCCGCTTCCAGCTCTATCTGCGGCAGGCACGGGCTGTGTGCACCCAG 1320

DT40 1321 TCCTGGGCCCGCTTTCATCCTCTGCGTATGGTGGGGGGCTGCACCCTCATTGCTGCTTCC 1380

||||||||||||||||||||||||||||||||||||||||||||||||||||||||||||

Refe 1321 TCCTGGGCCCGCTTTCATCCTCTGCGTATGGTGGGGGGCTGCACCCTCATTGCTGCTTCC 1380

DT40 1381 TGCTTGCTCTGCTATGTGGCCTCAGAGCTGGCCACAGTGTCAGACTCTTTCTATCGCAGC 1440

||||||||||||||||||||||||||||||||||||||||||||||||||||||||||||

Refe 1381 TGCTTGCTCTGCTATGTGGCCTCAGAGCTGGCCACAGTGTCAGACTCTTTCTATCGCAGC 1440

DT40 1441 TGCCTCCTGTACCCATTGCTTTGGGGGCTGGTGACAGCTGTTCTGCGTGGCCTAGCCTGT 1500

||||||||||||||||||||||||||||||||||||||||||||||||||||||||||||

Refe 1441 TGCCTCCTGTACCCATTGCTTTGGGGGCTGGTGACAGCTGTTCTGCGTGGCCTAGCCTGT 1500

DT40 1501 GTGTTCACCCAAGAGGAGCTGGATCTCCTCCTGCTGTTGTCTTGGGCAGCTGCAGCATCT 1560

||||||||||||||||||||||||||||||||||||||||||||||||||||||||||||

Refe 1501 GTGTTCACCCAAGAGGAGCTGGATCTCCTCCTGCTGTTGTCTTGGGCAGCTGCAGCATCT 1560

DT40 1561 CTGCTGGGCTTTTTCTGGCACTGGTGGGGCAGGCATCCCAAGCGAGCCCGTTTGTTGGGC 1620

||||||||||||||||||||||||||||||||||||||||||||||||||||||||||||

Refe 1561 CTGCTGGGCTTTTTCTGGCACTGGTGGGGCAGGCATCCCAAGCGAGCCCGTTTGTTGGGC 1620

DT40 1621 AGCCAACCACCCTTGGCCAGCATTGGTCTGAGGCAGAGGCTGCGGGTGTGGCTTGGGCTG 1680

||||||||||||||||||||||||||||| ||||||||||||||||||||||||||||||

Refe 1621 AGCCAACCACCCTTGGCCAGCATTGGTCTAAGGCAGAGGCTGCGGGTGTGGCTTGGGCTG 1680

DT40 1681 GCCTTCCCCGTGAGCATTCTCTTTTTCCGCTGCGGAGCTATGTTCTCTGATAGCTTTGTG 1740

||||||||||||||||||||||||||||||||||||||||||||||||||||||||||||

Refe 1681 GCCTTCCCCGTGAGCATTCTCTTTTTCCGCTGCGGAGCTATGTTCTCTGATAGCTTTGTG 1740

DT40 1741 GTAGCTGAGGCCCAAGTAGCCCCATTCCTGTTGGCCTCACTGGTGATGTTGCTGATAGGG 1800

||||||||||||||||||||||||||||||||||||||||||||||||||||||||||||

Refe 1741 GTAGCTGAGGCCCAAGTAGCCCCATTCCTGTTGGCCTCACTGGTGATGTTGCTGATAGGG 1800

DT40 1801 AAGCTCCACTGGGATGGTCACCTGACTGTGCCAGAAGGCCCCAAACAGCAGGCTCTTGGC 1860

||||||||||||||||||||||||||||||||||||||||||||||||||||||||||||

Refe 1801 AAGCTCCACTGGGATGGTCACCTGACTGTGCCAGAAGGCCCCAAACAGCAGGCTCTTGGC 1860

DT40 1861 TTTTCCTCTTACCGAAAAGAGAGCTGGCACCTGCTGTGCCTTGTGGCTGTGCTTCTGGTT 1920

||||||||||||||||||||||||||||||||||||||||||||||||||||||||||||

Refe 1861 TTTTCCTCTTACCGAAAAGAGAGCTGGCACCTGCTGTGCCTTGTGGCTGTGCTTCTGGTT 1920

DT40 1921 TGTGTGCGACTCTCTGGTTTCTTCCACCAGTGCCGTGAAGAAATCCCTCAGTGCCGGCCC 1980

||||||||||||||||||||||||||||||||||||||||||||||||||||||||||||

Refe 1921 TGTGTGCGACTCTCTGGTTTCTTCCACCAGTGCCGTGAAGAAATCCCTCAGTGCCGGCCC 1980

DT40 1981 TCCGTTTTCCTTCCACCTCTTGCCAGCCTGAGAAACACACGGGCCAAGAACCTCTTCTAC 2040

||||||||||||||||||||||||||||||||||||||||||||||||||||||||||||

Refe 1981 TCCGTTTTCCTTCCACCTCTTGCCAGCCTGAGAAACACACGGGCCAAGAACCTCTTCTAC 2040

DT40 2041 CTCTTATGTGTGGCCTTGCTGGCTGGGCTGGTGTATGCAGTGCGGAGCTGGCTGCGTCAC 2100

||||||||||||||||||||||||||||||||||||||||||||||||||||||||||||

Refe 2041 CTCTTATGTGTGGCCTTGCTGGCTGGGCTGGTGTATGCAGTGCGGAGCTGGCTGCGTCAC 2100

DT40 2101 TATGGCAATCTGAACAGCTCAGACCCCCTTGTGCTCTTTGTGCGCTGGGGTTTCCCACTG 2160

||||||||||||||||||||||||||||||||||||||||||||||||||||||||||||

Refe 2101 TATGGCAATCTGAACAGCTCAGACCCCCTTGTGCTCTTTGTGCGCTGGGGTTTCCCACTG 2160

DT40 2161 GTGGTCCTTTGCATCGCCTGCTACTGGGCTGTTGCCTCCAGTGCTGAGGACTCTCTGGGC 2220

||||||||||||||||||||||||||||||||||||||||||||||||||||||||||||

Refe 2161 GTGGTCCTTTGCATCGCCTGCTACTGGGCTGTTGCCTCCAGTGCTGAGGACTCTCTGGGC 2220

DT40 2221 AAGCTGCAGGAGCTGGTAGAGGTGGCAATCATTGCCTTTCCATGGGCTGTCTATGGGTTA 2280

||||||||||||||||||||||||||||||||||||||||||||||||||||||||||||

Refe 2221 AAGCTGCAGGAGCTGGTAGAGGTGGCAATCATTGCCTTTCCATGGGCTGTCTATGGGTTA 2280

DT40 2281 GTGTCTATTGGGTTGCTGCTCCTGCTGTGCCATCCCATGACAGTGTTTGCAAAGGACTCA 2340

||||||||||||||||||||||||||||||||||||||||||||||||||||||||||||

Refe 2281 GTGTCTATTGGGTTGCTGCTCCTGCTGTGCCATCCCATGACAGTGTTTGCAAAGGACTCA 2340

DT40 2341 CGGGAATCTGCAGGATCCATTGTCACTCCCTACCAGGGGATTCCCAGCTCCAAAGTCGAC 2400

||||||||||||||||||||||||||||||||||||||||||||||||||||||||||||

Refe 2341 CGGGAATCTGCAGGATCCATTGTCACTCCCTACCAGGGGATTCCCAGCTCCAAAGTCGAC 2400

DT40 2401 TTGCTCCAGGTCATCCCTCAGATCTACAAGAGGATGCAGGAGTCTGAGAAGAGTCGCCTG 2460

||||||||||||||||||||||||||||||||||||||||||||||||||||||||||||

Refe 2401 TTGCTCCAGGTCATCCCTCAGATCTACAAGAGGATGCAGGAGTCTGAGAAGAGTCGCCTG 2460

DT40 2461 GAACGGCGCAGCTGCAGGGCCACAGTTGCAGCCTATGGGCTAGGCAGCGTGTACTCGGCA 2520

||||||||||||||||||||||||||||||||||||||||||||||||||||||||||||

Refe 2461 GAACGGCGCAGCTGCAGGGCCACAGTTGCAGCCTATGGGCTAGGCAGCGTGTACTCGGCA 2520

DT40 2521 GCCCTGGTCATAGCACTCACCCTCCTGGGCTTCCTCTTGATGCTCCTGCACAGTGAGCGG 2580

||||||||||||||||||||||||||||||||||||||||||||||||||||||||||||

Refe 2521 GCCCTGGTCATAGCACTCACCCTCCTGGGCTTCCTCTTGATGCTCCTGCACAGTGAGCGG 2580

DT40 2581 CTCAGTCTTGCCTTCCTACTCCTCTTCCTGGAGGCCTTTGTGCTGCTGCACATCCACACA 2640

||||||||||||||||||||||||||||||||||||||||||||||||||||||||||||

Refe 2581 CTCAGTCTTGCCTTCCTACTCCTCTTCCTGGAGGCCTTTGTGCTGCTGCACATCCACACA 2640

DT40 2641 CGTGCCAGAAGCCTTGCAGGAGACACTGAGCCTTTTTCAGTGCCCTGGTTTTCAGTCATC 2700

| ||| | | | | || | || ||||||||||||||||||||||||||||||

Refe 2641 CCAGCC-G---C-T---A--AGCC-CTCT-CCTTTTTCAGTGCCCTGGTTTTCAGTCATC 2688

DT40 2701 TCGTGGCTCCTTGCTGCTTCTCAGTTCTTCTATTCCACGGGCCATCAGCCCATCTTCCCG 2760

||||||||||||||||||||||||||||||||||||||||||||||||||||||||||||

Refe 2689 TCGTGGCTCCTTGCTGCTTCTCAGTTCTTCTATTCCACGGGCCATCAGCCCATCTTCCCG 2748

DT40 2761 GCCATCCACTGGAATGCAGCTTTTGTGGGCTTTCACCTTGACCACAGCACAAACCTCCTT 2820

||||||||||||||||||||||||||||||||||||||||||||||||||||||||||||

Refe 2749 GCCATCCACTGGAATGCAGCTTTTGTGGGCTTTCACCTTGACCACAGCACAAACCTCCTT 2808

DT40 2821 CCTGCTGTCCTGGTGGGCGCCAACACGTTTGCCTCCCATATCCTCTTTGCAGTTGGCTGC 2880

||||||||||||||||||||||||||||||||||||||||||||||||||||||||||||

Refe 2809 CCTGCTGTCCTGGTGGGCGCCAACACGTTTGCCTCCCATATCCTCTTTGCAGTTGGCTGC 2868

DT40 2881 CCTCTGCTCCTGCTTTGGCCCTTTGTGTGTGAGATGCCCAGCTCAGAGAAGAAGAAGTCC 2940

||||||||||||||||||||||||||||||||||||||||||||| ||||||||||||||

Refe 2869 CCTCTGCTCCTGCTTTGGCCCTTTGTGTGTGAGATGCCCAGCTCACAGAAGAAGAAGTCC 2928

DT40 2941 AAGAGGGAGCCTCAGGAGGAGCTGCAGACAGTGGATGAACACATGATGGAGATGAGGCTG 3000

||||||||||||||||||||||||||||||||||| ||||||||||||||||||||||||

Refe 2929 AAGAGGGAGCCTCAGGAGGAGCTGCAGACAGTGGACGAACACATGATGGAGATGAGGCTG 2988

DT40 3001 CGGGAGTCCCCA 3012

||||||||||||

Refe 2989 CGGGAGTCCCCA 3000
